# An Approach to Improve Dementia Health Literacy in Indigenous Communities

**DOI:** 10.1007/s10823-019-09388-2

**Published:** 2019-12-18

**Authors:** Sharlene Webkamigad, Wayne Warry, Melissa Blind, Kristen Jacklin

**Affiliations:** 1 School of Rural and Northern Health, Laurentian University, Sudbury, ON Canada; 2 University of Minnesota, Medical School Duluth, 624 E 1st Street, Unit 201, Duluth, MN 55805 USA

**Keywords:** Cultural safety, First Nations, Health promotion, Indigenous knowledge, Knowledge translation, Two-eyed seeing

## Abstract

This project aims to improve health literacy in Indigenous communities through the development of evidence-based culturally relevant health promotion materials on dementia that bridge the gap between Indigenous and Western perspectives of the illness. The research team worked in partnership with Health Canada’s First Nations and Inuit Home and Community Care Program (FNIHCC) and consulted with Indigenous elders to utilize a two-eyed seeing framework that draws upon Indigenous knowledge and Western biomedicine. A consolidated review of materials and research involving Indigenous perspectives of Alzheimer’s and age-related dementias led to the development of two culturally appropriate fact sheets. Two Indigenous-specific fact sheets were developed “What is Dementia? Indigenous Perspectives and Cultural Understandings” and “Signs and Symptoms of Dementia: An Indigenous Guide.” The fact sheets prioritize Indigenous knowledge and pay particular attention to Indigenous languages, diverse Indigenous cultures, and literacy levels. The content uses phrasing and words from Indigenous people involved in the research to share information. Biomedical concepts and words were included when necessary but language or presentation of these aspects were often modified to reflect Indigenous conceptualizations. This project provides a foundation for evidence-based knowledge translation in relation to cultural safety in dementia care. Specifically, the researchers outline how health care providers can develop culturally appropriate health promotion material, thus increasing Indigenous cultural understandings of dementia and health literacy.

There are roughly 1.4 million Indigenous people^1^ in Canada (Statistics Canada 2013), which includes First Nations, Inuit, and Métis (Health Council of Canada 2012). There are 618 First Nations in Canada, with distinct values, beliefs and language dialects (Aboriginal Affairs and Northern Development Canada 2015). There have been reports that Indigenous people experience the poorest health of any group of people in Canada (Health Council of Canada 2012; Royal College of Physicians and Surgeons of Canada 2013); however, these reports are fragmented and do not account for the ways in which Indigenous people perceive health (Loppie Reading and Wien 2009). Social determinants of health such as structural violence, racism, and discrimination are amongst the socio-economic health disparities (Allan and Smylie 2015) and have led to chronic health conditions experienced by Indigenous peoples at higher than then average rates, which also increases their risk of developing dementia (Walker and Jacklin 2019). Dementia is an umbrella term for a variety of brain disorders with symptoms such as memory loss, problems with judgement and reasoning, and changes in mood and behaviour, and these symptoms include issues with brain function that can affect a person’s ability to function at work, in relationships, or in everyday activities (Alzheimer Society of Canada 2012).

Similar to the non-Indigenous population, the Indigenous older-adult age group (age 65 years and over) is projected to increase rapidly. There is a projected growth of the Indigenous identity population in Canada aged 65 years and over from 4.7% in 2006 to an expected 14.5–15.4% by 2031 (Malenfant and Morency 2011). Increasing age is the most significant irreversible factor in the development of dementia. There have been very few studies that address the rates of dementia in Indigenous people (Warren et al. 2015); however a recent study suggests that the age-standardized prevalence of dementia in First Nations in Alberta in 2009 was 7.5 per 1000, compared to non-First Nations, at 5.6 per 1000 (Jacklin et al. 2013a). Moreover, Walker and Jacklin suggest that there will be a fourfold increase in the number of First Nations people over age 60 with dementia in 2031, compared to non-First Nations population, who will have a 2.3-fold increase (Walker and Jacklin 2019).

Research shows a lack of basic dementia care resources in Canada, especially Indigenous-specific dementia care literature (Finkelstein et al. 2012). Caregivers, older adults, nurses, personal support workers, and researchers often request Alzheimer’s and age-related dementias health promotion materials targeted to the Indigenous peoples in Canada but none exist (Jacklin et al. 2014b; 2014c Pace et al. 2013). The purpose of this article is to outline a unique and innovative process resulting in two evidence-based Indigenous-specific fact sheets on dementia care for caregivers, older adults, people with dementia, and community health care providers.

## Background

Canadian literacy scores are generally lower for Indigenous people and older adults, and 60% of adults and 88% of older adults are thought to not have adequate levels of health literacy (Chiarelli and Edwards 2006; Public Health Agency of Canada 2014). Health literacy is defined as an individual’s ability to access, comprehend, evaluate, communicate, and use health information and services as a way to promote, maintain, and improve health and make good health-related decisions in a variety of settings across the life-course (Korhonen 2006; Nielsen-Bohlman et al. 2004; Public Health Agency of Canada 2014; Rootman and Ronson 2005; Vass et al. 2011). Furthermore, literacy, one of the 12 determinants of health (Public Health Agency of Canada 2013), “is constructed and enacted within social, cultural, and political contexts” (Smylie et al. 2006, p. S22).

Increased understandings of health disparities has led to an ongoing priority amongst policy makers to improve health literacy for Indigenous peoples (Chiarelli and Edwards 2006; Smylie et al. 2006). Yet, very few studies explain how Indigenous approaches to health literacy are utilized (Vass et al. 2011). Health care providers can address cultural safety and health literacy by considering the languages, cultural and social influences, education levels, reading skills, language-comprehension skills, listening skills, background knowledge and concepts of health-related topics, numeracy skills, emotional and physical factors, and the individual’s level of comfort in the health setting (Korhonen 2006). The researchers of this paper anticipate that addressing access to health literacy for Indigenous people will improve health care and help reduce the effects of structural barriers and improve cultural safety.

Cultural safety, a model originally developed by Dr. Irihapeti Ramsden, highlights how colonial, historical, and sociopolitical factors led to health disparities in Indigenous communities (Allan and Smylie 2015). Placing equal focus on both Indigenous knowledge and Western biomedicine enforces cultural safety in that it responds to the inequities experienced by Indigenous people.

Decolonizing health information to improve health literacy requires an understanding that there are separate belief systems within different cultural groups that may contrast with Westernized biomedical beliefs (Nielsen-Bohlman et al. 2004), and requires the efforts of health professionals to understand the culture and everyday experiences of Indigenous peoples (Jacklin and Warry 2012). Indigenous people in North America are recognized as holding culturally specific understandings of dementia that influence health care seeking behaviour and caregiving (Henderson and Henderson 2002; Jacklin and Warry 2012; Lanting et al. 2011). In many cases, Indigenous peoples connect memory loss and confusion associated with aging as “normal,” “natural,” and “accepted” (Jacklin et al. 2015a), or “supernormal” (Henderson and Henderson 2002). Dementia knowledge associated with Western biomedical definitions may also be present (Hulko et al. 2010).

Culture, life experiences, and religious beliefs may affect Indigenous peoples understanding of aging, dementia, and perceptions of the caregiving role (Hayter et al. 2008; Luong et al. 2014). Pace et al. (2013) argues that an appropriate understanding of personhood is important when developing culturally safe dementia care such as fact sheets. Furthermore, a discussion of the concepts of person-centred language and personhood is argued to be necessary to maintain the respect and recognition towards Indigenous people, older adults, and caregivers. There are variations in the form and function of personhood: egocentric (personal history, accomplishments), sociocentric (family, clan, lineage, community), ecocentric (environment, ecology), and cosmocentric (ancestors) cultural configurations of the self (Kirmayer 2007). The Alzheimer Society (2012) puts forth personhood as the first principle to support person-centred language:

“A standing or status that is bestowed upon one human being by others in the context of relationship and social being. It implies recognition, respect and trust” (Alzheimer Society of Canada 2010, p. 2).

The concepts of person-centred care and personhood are potentially problematic when applied to the Indigenous context. Person-centred care and personhood are egocentric, focus on the individual, and may contradict with relational Indigenous ways of life. Care for older adults in the Indigenous context is ideally provided by the family and grounded in traditional values (Jacklin et al. 2015b), thus supporting a dynamic, relational view (Morhardt and Spira 2013) grounded in values of collectivism, love, reciprocity, and the understanding of the circle of life. Peoplehood, a concept developed in the 1980’s by Robert K. Thomas, identifies four factors that imply how Indigenous people behave: language, sacred history, ceremonial style (formerly religion), and land (Holm et al. 2003). Understanding the applicability of concepts of peoplehood, personhood, and person-centred care can help to determine the information that is given in dementia health promotion fact sheets. In this paper, the context of peoplehood is preferred as it supports an ideology of relational, sociocentric care.

## Methods

Indigenous dementia fact sheets were developed using a decolonizing framework known as two-eyed seeing (Estey et al. 2008; Martin 2012) and were guided by a cultural safety approach (Gerlach 2012; Hart-Wasekeesikaw 2009). Two-eyed seeing is a framework proposed by Mi’Kmaw elders Albert and Murdena Marshall (Martin 2012), who suggest that two-eyed seeing is understanding, acknowledging, and respecting a diversity of perspectives of the world “without perpetuating the dominance of one over the other” (Martin 2012, p. 24). We have used this approach as a unique method to develop health promotion material that combines Indigenous and Western knowledge of dementia. To ensure appropriate knowledge translation strategies, the process included: 1) an analysis of Indigenous beliefs about dementia from a previous foundational research study, 2) an academic literature review, 3) an environmental scan of health promotion resources, and, significantly, 4) on-going consultations with various Indigenous community health leaders, collaborators, and experts and continuing consultation with an Indigenous knowledge keeper.

The research team began with an analysis of findings from a previous community-based participatory research study “Perceptions of Alzheimer’s Disease and Related Dementias among Aboriginal Peoples in Ontario” – in this paper referred to as the Perceptions Project. This study focused on the perspectives of dementia in six communities in Ontario, Canada (Jacklin et al. 2014a, 2014b, 2014c; Jacklin et al. 2013c; Pace et al. 2013). The communities include Sudbury, Thunder Bay, Ottawa, Six Nations, Moose Cree First Nation, and a combined group of Anishinaabek First Nations on Manitoulin Island. The purpose of the Perceptions Project was to engage both male and female Indigenous people with dementia, their caregivers, health care providers, Indigenous knowledge keepers and other key informants to gain diverse knowledge about the attitudes, beliefs, and behaviours related to dementia. The authors reanalyzed the data focusing on early warning signs/symptoms of dementia and cultural understandings of dementia with the intent of transitioning the findings into various health promotion materials. Drawing on the data specifically for the creation of the fact sheets, the location of the interviews, participant codes, key ideas, key quotes and notes were captured in data analysis tables for each community. In addition, the authors discussed the crucial themes from each location and then consolidated the data into two tables. One table focused on Fact Sheet #1: the cultural understandings of dementia, while the other covered Fact Sheet #2: the early warning signs and symptoms.

The next step involved reviewing published literature on dementia in Indigenous populations. In this step the authors were interested in ensuring the findings from the Perceptions Project would have national relevance and be applicable in other provinces and First Nations. The authors drew upon and updated an existing literature review previously prepared in 2012 by one of the authors for First Nations and Inuit Health (Jacklin and Walker 2012). The literature review was updated to include references through 2016.

The third step involved an environmental scan of relevant unpublished web-based documents and paper-based resources. The authors extracted findings that were relevant to the health promotion materials related to dementia in Indigenous peoples in Canada, the United States, New Zealand, and Australia. The environmental scan also allowed a review of specific health promotion material on various topics (e.g., diabetes, cancer) developed for Indigenous people in Canada to assess for readability, design, visuals, color, and the overall presentation of the information.

Finally, collaborations were a key mechanism to ensure appropriateness and cultural safety. On-going collaboration between the principal investigators (KJ, WW) with Health Canada’s First Nations and Inuit Home and Community Care program (FNIHCC) occurred throughout the project. Stakeholders included First Nations FNIHCC program managers from across Canada. The principal investigators and FNIHCC program staff shared the common goal of improving the cultural relevance of materials and programming in the area of Indigenous dementia care. The collaboration involved shared financial contributions, an on-going dialogue on the research process and outcomes as well as dissemination strategies. The collaborative approach included two methods: (1) to facilitate the inclusion of a policy and programming perspective the PIs met bi-monthly with program staff from the FNIHCC office, and (2) during the final month of the project the principal investigators were invited to the FNIHCC program community stakeholders meeting where the two fact sheets were presented and discussed in relation to their applicability at a national level. Significantly, the two-eyed approach fostered a close collaboration with a project elder, Jerry Otowadjiwan, to develop and refine the resources. Elder Otowadjiwan is a life-long resident of Manitoulin Island, Anishinaabemowin language specialist, Fourth Degree Midewin, and Mishomis, and he provided guidance regarding the medicine wheel and life cycle models (Jacklin et al. 2017d).

This project is unique in that two-eyed seeing helped to combine Indigenous with biomedical knowledge to develop the content in the fact sheets. This is accomplished by using the findings from the community reports to populate the fact sheets, followed by integrating published literature as appropriate. The environmental scan identified best practices in health promotion, as well as gaps in dementia materials aimed at Indigenous people in Canada. The authors relied on consultations with FNIHCC partners and Elder Otowadjiwan to strengthen and verify the content and language used was culturally appropriate and relevant. Lastly, the project team reviewed drafts of the fact sheets with consideration for recommendations from an Alzheimer Society of Canada (ASC) document titled “Person-Centred Language” (Alzheimer Society of Canada 2010) and sought a literacy level of five using the Flesch-Kincaid Index feature on Microsoft Word (described in results). Special attention was given to word selection, complex sentences, and sentence structure.

## Results

This multi-stage knowledge translation process resulted in two polished fact sheets titled “What is dementia? First Nations Perspectives and Cultural Understandings”, and “Signs and Symptoms of Dementia: A First Nations Guide” (http://www.i-caare.ca/factsheets). The material incorporated biomedical information with Indigenous knowledge for a population of older Indigenous adults in Canada. Between the launch of the material in April 2017 to July 2018, the web domain http://www.i-caare.ca/factsheets received over 7000 visits. Hard copy distribution is not supported through the research funding, however, the authors have been able to distribute over 2500 hard copies upon request to health care providers across Canada and approximately 400 hard copies to Tribal Nations in Minnesota and Wisconsin, USA. Providers and organizations requesting hard copies of the fact sheets include provincial health authorities, local chapters of the Alzheimer’s society, First Nations/Tribal communities, and Indigenous health centers from across Canada.

### Fact Sheet 1: What Is Dementia? First Nations Perspectives and Cultural Understandings

The findings from the Perceptions Project and the literature review found that Indigenous cultural and Western understandings of dementia differ. Most Indigenous people accept memory loss and confusion as a normal part of the aging process and these understandings are intimately connected to Indigenous values and worldviews (Hulko et al. 2010; Jacklin et al. 2014a, 2014b, 2014c; Jacklin et al. 2013b; Jacklin et al. 2013c; Lanting et al. 2011; Pace et al. 2013). In the environmental scan, the authors found that many of the resources developed by Alzheimer’s organizations described dementia as an irreversible disease affecting the brain, causing deterioration of memory and the ability to think; and is also described as causing behavior and emotional changes that interfere with the person’s social and work life (Alzheimer Society of Canada 2010). Both Indigenous and Western perspectives were respected by developing a description of the meaning of dementia while highlighting the Indigenous perspective. This excerpt is taken from the fact sheet:

There is some evidence that suggests age-related dementias have only recently become more common in Indigenous populations. As people live longer, they are more likely to experience dementia. Just as First Nations communities in Canada are different, First Nations peoples, communities and cultures hold different understandings of dementia, memory loss, forgetfulness and confusion related to aging. These understandings may be very different from those held by doctors, nurses and support workers. Some descriptions of dementia that are common are that: “it’s normal”, “it’s natural”, “it’s part of the circle of life” or “coming full circle”. Dementia may also be described as a “second childhood” and a time when one is “closer to the Creator.” A person’s spiritual beliefs often influence how dementia is viewed (Jacklin et al. 2017b).

The results suggested that the term dementia may or may not be an accepted or conceptually understood term for all Indigenous people (Hulko et al. 2010; Sutherland 2007), and that there may be some underlying shame or stigma associated with the disease (Pace et al. 2013). Furthermore, there is an absence of the word “dementia” in Indigenous languages. There are, however, Indigenous words that describe the symptoms or state of mind associated with memory loss. For example, the Tahltan First Nations people in British Columbia say, “Kadousah”, which means “not knowing if you’re coming or going”; or “Edu M’Diid Sugo ta a”, which means “not brain well” (Stevenson 2014). Similarly, participants from the Perceptions Project shared translated Indigenous phrases such as “my mind goes on me”, “breakdown of thoughts”, “sickness of the mind”, “brain not working right”, and “losing one’s mind”, which were used to describe the loss of balance between the mind, the body, and spirit (Jacklin et al. 2014a; Jacklin et al. 2013b). In the two fact sheets, an incorporation of words and phrases that came from the Perceptions Project appeared to be harmonious across the provinces according to the literature available on the topic. Language issues in the fact sheets were addressed as follows:

Dementia may or may not be an accepted term for all people. It may be more appropriate to speak of forgetfulness or thoughts being mixed up. There is no word that has been identified to mean dementia in Indigenous languages in Canada. Instead, First Nations languages have words that describe the symptoms or state of mind. For example, words and phrases such as: “forgetful”, “confused”, “thoughts mixed up”, “something wrong with my head”, “mind changes”, “going back to childhood” (Jacklin et al. 2017b).

Both the Perceptions Project and literature review found that colonialism and racism remain pervasive issues in the lives of Indigenous people in Canada, often resulting in some individuals being “out of balance” physically, emotionally, mentally, or spiritually. It has been documented that changes to Indigenous lifeways and culture have changed conceptions of dementia and aging overtime (Hulko et al. 2010; Lanting et al. 2011; Pace 2013; Wilson et al. 2011). These changes include collective suffering and historical trauma from the consequences of colonization and loss of connection to the land (Arkles et al. 2010; Mundel and Chapman 2010; Wilson et al. 2011). The resulting shift from a traditional way of life is expressed as a significant risk factor for those elders who experience these challenges. The following is the excerpt from the fact sheet that responds to this finding:

Historical changes in diet, changes to the land or environment, disconnection from culture, as well as trauma, intergenerational trauma, stress, and unresolved grief are significant factors that cause people and communities to sometimes be out of balance and may partially explain a rise in the number of elderly with dementia (Jacklin et al. 2017b).

### Fact Sheet 2: Signs and Symptoms of Dementia: A First Nations Guide

The review of materials suggests that Indigenous people are reluctant to discuss common signs and symptoms with their health care providers (Durey et al. 2012; Stevenson 2014). With the goal of acknowledging the main factors contributing to this reluctance and in an effort to encourage more active involvement with health systems at earlier stages, the authors included this explanation in the fact sheet:

Some First Nations people may be reluctant to discuss forgetfulness or memory loss with health care providers for many reasons, for example: they consider it to be a normal part of aging and not problematic, they have not had good experiences with health care providers in the past, they do not think a diagnosis would make any difference, they are embarrassed and do not want others to know about their symptoms, they do not want to take the memory tests, or they have difficulty accessing health care services (Jacklin et al. 2017a).

Many Indigenous people perceive memory loss to be a normal part of the life cycle (Henderson and Henderson 2002; Hulko et al. 2010; Lanting et al. 2011), and the analysis of the literature suggested that as in other populations, Indigenous people often asked or wondered where the line is between normal forgetting and illness-based forgetting (Pace et al. 2013). The authors examined how other health promotion materials dealt with this issue and adapted the language to reflect the differences. Meaningful differences of when memory loss is normal and when it is a symptom of illness using Indigenous specific experiences are included in the fact sheet and described in Table 1.

**Table 1:** When forgetfulness is part of normal aging versus a symptom of illness

Normal Aging	Symptom of Illness
Forgetting can be a normal part of getting older. We may forget to pay a bill, lose things, and find it hard to remember the words we want to use every once and a while. It can be normal for our memories of events to be less detailed than they once were, we may take a little bit longer to remember. Sometimes we may have words on the tips of our tongues that we cannot find in the moment.	When these types of forgetting become worse over time or begin to happen more often, it may be a sign that something is wrong. For example, missing one bill payment once and a while may be normal, but missing many payments and not being able to manage money may not be normal. Losing track of the day may be normal, but losing track of the month or season is not. Difficulty finding the words we want may sometimes be normal, but not being able to carry on a conversation is not.

Reproduced from Jacklin et al. 2017a

The contrast between mainstream messages and Indigenous messages about common elements of signs and symptoms of dementia are listed in Table 2 (Alzheimer’s Association 2015; Alzheimer’s New Zealand 2012; Australia Alzheimer’s 2010; Canadian Alzheimer’s Society 2015).

**Table 2:** Examples contrasting the language of early warning signs and symptoms of dementia

Mainstream messaging	Indigenous messaging
Problems with language, words, speaking, writing, and understanding what is said to them	Forgetfulness, including not being able to recognize people, places, or objects; repeating stories
Difficulty performing/completing/managing familiar complex or new tasks and taking longer to do so	Impaired judgement, including not understanding instructions; not understanding cause and effect
Withdrawal from usual/social/work activities due to lack of initiative or loss of social skills	Withdrawn from family, friends, or favourite activities
Changes in mood, behavior and personality with unpredictable behavior and ‘mood swings’	Changes in mood and personality, including emotional outbursts; changes in hygiene, including forgetting to brush hair, teeth; not interested in washing or getting cleaned up
Challenges in planning or solving problems, with impaired/decreased/poor judgement	Easily upset or frustrated; increased fear, paranoia or distrust of others; compulsive behaviour
Confusion with time or place/disorientation in time and space	Getting lost, this may include wandering or pacing; seeing or hearing things that nobody else can, including connections with people who have passed; hallucinations; delusions; sense of time is off
Misplacing things	Misplacing objects

Reproduced from Jacklin et al. 2017a

An analysis of the Perceptions Project data showed that Indigenous perspectives of signs and symptoms of dementia shared some common elements with what was found in the environmental scan and literature review (Henderson and Henderson 2002; Lampton 1998; Smith et al. 2011). For example, through the Perceptions Project, it was found that some Indigenous people perceive seeing or hearing things that nobody else can, including deceased family members, as a normal part of growing older (Jacklin et al. 2014c). Hallucinations may not be problematized by Indigenous people as they can be perceived as actual communications with the supernatural, or spiritual, world (Henderson and Henderson 2002). However, recognizing the need to respect the Indigenous interpretations of these events with the potential of serious medical complications, the fact sheet identifies that there may be hallucinations that are not natural:

Hearing or seeing things that other people cannot may be a symptom of a more serious acute condition related to severe infection or the effects of prescription medications. If you think you may be experiencing unnatural hallucinations, you should seek care immediately (Jacklin et al. 2017a).

The Alzheimer Society of Canada document “Person-Centred Language” was determined to conflict with core Indigenous values. The document suggested avoiding the term “loved one(s)” when referring to the person with dementia. It is suggested this term is challenging because relationships between family members may be difficult, some may be uncomfortable with the expression, and that it is funereal in tone (Alzheimer Society of Canada 2010). The guideline recommends using “person/people with dementia”, “family member”, or “friend” instead.

To reflect language of Indigenous peoples, Indigenous knowledge was pursued. Love, one of the Seven Grandfather Teachings, is an essential component of care provided to a person with dementia. The action of love is giving part of yourself to another. Following discussions with Elder Otowadjiwan, the authors identified that the term “loved one” is commonly accepted and welcomed in the Indigenous context as caring for a person with dementia requires a lot of love, understanding and patience (Jacklin et al. 2015b). As a result, the term “loved one” was used in the fact sheet.

## Discussion

The researchers in this study supported Indigenous and Western understandings of dementia in the development of the fact sheets by utilizing a two-eyed seeing approach to decolonize health information. By embracing a broad knowledge translation approach in gathering information regarding dementia, the researchers have strengthened the pathway in decolonizing health information by combining knowledge that leads to a new perspective gained by seeing through two eyes. Two-eyed seeing reflects an ability to view strengths of both Indigenous and Western biomedical knowledge and ways of knowing (Estey et al. 2008). This framework has offered a unique and innovative way of uniting key ideas gained from consulting with Indigenous elders and scholars, evidence-based findings from the Perceptions Project and scholarly literature, and the contribution of both Indigenous and biomedical perspectives.

Part of the knowledge translation process included focussing on common health promotion considerations such as literacy level and language choice. The authors found throughout the knowledge translation process that high-syllable words greatly affected the readability level of the contents of the fact sheets. Special attention was therefore given to word selection, complex sentences, and sentence structure. The literacy level was assessed using the Flesch-Kincaid Index feature on Microsoft Word. The Flesch-Kincaid grade level of the “What is dementia? First Nations Perspectives and Cultural Understandings” and “Signs and Symptoms of Dementia: A First Nations Guide” fact sheets were 12.6 and 11.3, respectively. A Flesch-Kincaid grade level of 5 was sought throughout the materials, however the inclusion of terms such as Alzheimer’s, dementia, and Indigenous greatly impacted the readability level. Even when adjusting the fact sheet to exclude high-syllable words such as dementia(s), Alzheimer’s, Indigenous, understanding(s), and community, the Flesch-Kincaid grade level was reduced to 11.2 and 10.5. The literature suggests different strategies to ensure the material is presented to counter low literacy levels when delivering health information (Jones et al. 2013) and special attention should be paid to future health promotion material that includes high-syllable words.

Throughout the development of the fact sheets, the authors identified differences between acceptable and unacceptable words used in the fact sheets (i.e. loved one). As a result, language choice became an important dialogue. As the authors utilized a person-centred care resource during the development of the fact sheets (Alzheimer Society of Canada 2012), it was also necessary to consult with the project Elder regarding terms that are egocentric in tone and which may not be acceptable in an Indigenous context. It was determined that elements from personhood and person-centred care can be used to identify how to deliver care to the individual (Kirmayer 2007; Morhardt and Spira 2013); however, this approach should be adapted further to include other aspects of the support system (e.g. loved ones, elders, community members). Language supportive of peoplehood and an understanding of relational care provided by the family can be used to ensure the unique values of Indigenous peoples are encouraged (Holm et al. 2003; Jacklin et al. 2015b). Therefore, it is more applicable to utilize terms that are congruent with an understanding of peoplehood as this resembles Indigenous values of caregiving.

It is important that health professionals in the field of health literacy, health promotion, and Indigenous knowledge translation understand local socio-cultural realities to truly reflect culturally safe material. These recommendations are congruent with other researchers’ findings (Lindeman et al. 2010; Smylie et al. 2006, 2009). When working within a culturally diverse population, health care professionals must understand that there are differences in intercultural communication amongst Indigenous peoples (Jacklin et al. 2017c; Smylie et al. 2009; Taylor et al. 2012).

The collaborative approach with FNIHCC facilitated a dialogue with diverse First Nations Home and Community Care program workers and managers. As an organization, the FNIHCC program had an interest in materials that were generic enough in design that they could be easily adapted for various First Nations based on their own culture. This resulted in the decision to provide the fact sheet content in two formats: (1) a version that could be modified to include locally appropriate and relevant content and (2) a second, polished version suitable for a national audience (http://www.i-caare.ca/factsheets). When the generic fact sheets were presented at the FNIHCC national stakeholder meeting there was widespread agreement with the content, the interpretation of the information sources, and that these perspectives were national in scope. The stakeholders further discussed the visual portrayal of the information. Some members commented that the information could be better portrayed in formats more accessible to them (e.g., a circle or medicine wheel); while others expressed concern for recognizing the diversity of Indigenous cultures and the need to consult the First Nation communities individually on the best visual representation. The stakeholder discussion reinforced the need for a generic approach to the content as well as the need to support diverse Indigenous communities in their uptake and modification of the materials based on local understandings of Indigenous systems of health knowledge.

Cultural safety is based on understanding inequities in health service delivery (Health Council of Canada 2012) and Indigenous approaches to health literacy (Smylie et al. 2006). To respond to the need for cultural safety in Indigenous health, this paper has enriched health care provider education. The authors are optimistic that sharing this approach will lead to systemic change in delivering culturally-safe care. The authors have also reinforced several of Korhonen’s recommendations of Indigenous approaches to health literacy by addressing cultural safety through considering the languages, reading skills, language-comprehension skills, background knowledge and concepts of health-related topics, and the individual’s level of comfort in the health setting (Korhonen 2006).

Health literacy improves the accessibility to information that has evidence-based Indigenous knowledge regarding memory loss and forgetfulness. Improving health literacy is thought to benefit both the individual and health system, in that it can improve self-management, allowing Indigenous people to make sound health decisions and reduce the use of health services (Mitic and Rootman 2012). Research suggests that older Indigenous people may be accessing services quite late in their dementia journey and are not benefiting from early detection and intervention (Buchignani and Armstrong-Esther 1999; Finkelstein et al. 2012; Henderson and Henderson 2002). The resultant fact sheets can serve to support those initial conversations between health care providers and Indigenous older adults and families concerned about memory loss and confusion; or, as a tool for individuals and families outside of the health system. In either case, the fact sheets hold the potential to raise awareness and lead to earlier engagement with health systems for memory loss and dementia. Indeed, the evidence available through the http://www.i-caare.ca website that hosts the fact sheets indicates that both mainstream and Indigenous health organizations are incorporating these tools into practice.

## Conclusion

The lack of culturally grounded health promotion material focused on dementia prompts the need for more attention in this area. Further action is needed within the health care sector to develop culturally-based health knowledge and to raise awareness of health concerns, and also to build capacity, infrastructure, and partnerships to improve health literacy and cultural safety for Indigenous peoples (Mitic and Rootman 2012). There is promising evidence through the number of requests our team receives that they are valuable resources that can be integrated into the portfolio of educational materials at organizations such as the Alzheimer Society of Canada. This project provides a foundation for evidence-based knowledge translation in relation to cultural safety in dementia care that draws heavily on a study that used a community based participatory action research approach and responds to community partner’s desire for action.

The approach outlined is one example of how knowledge translation in the Indigenous context must be deliberate in combining Western and Indigenous knowledge. The fact sheets use essential biomedical knowledge deemed important to convey Indigenous understandings and explanatory models of dementia. Two-eyed seeing paves the way for future Indigenous and non-Indigenous researchers, resource developers, and policy makers to provide culturally appropriate health promotion education and health literacy. The development of culturally appropriate health promotion materials for Indigenous communities requires a process to collect and analyze Indigenous knowledge on health-related topics; it is not simply a cut and paste process where mainstream materials are adapted through changes to imagery but not meaning. Improving the ability of Indigenous people to access, comprehend, evaluate, communicate, and use health information is essential to improving Indigenous peoples’ health.

## Footnotes

1 The term ‘Indigenous people’ will be used throughout this article when referring to the Aboriginal population (First Nations, Inuit, and Metis – original peoples of Canada) of Canada unless specifically referring to a First Nations group.
